# Chemotherapy reinforces anti-tumor immune response and enhances clinical efficacy of immune checkpoint inhibitors

**DOI:** 10.3389/fonc.2022.939249

**Published:** 2022-08-08

**Authors:** Lin Zhang, Chao Zhou, Songou Zhang, Xiaozhen Chen, Jian Liu, Fangming Xu, Wenqing Liang

**Affiliations:** ^1^ Department of Pharmacy, Shaoxing People’s Hospital, Shaoxing Hospital, Zhejiang University School of Medicine, Shaoxing, China; ^2^ Department of Orthopedics, Zhoushan Guanghua Hospital, Zhoushan, China; ^3^ College of Medicine, Shaoxing University, Shaoxing, China; ^4^ Department of Hepatobiliary Surgery, Shanghai Oriental Hepatobiliary Hospital, Shanghai, China; ^5^ Department of Gastroenterology, Zhoushan Hospital of Traditional Chinese Medicine Affiliated to Zhejiang Chinese Medical University, Zhoushan, China; ^6^ Medical Research Center, Zhoushan Hospital of Traditional Chinese Medicine Affiliated to Zhejiang Chinese Medical University, Zhoushan, China

**Keywords:** combination therapy, chemotherapy, immune checkpoint inhibitors, tumor microenvironment, cancer treatment

## Abstract

New evidence suggests that the clinical success of chemotherapy is not merely due to tumor cell toxicity but also arises from the restoration of immunosurveillance, which has been immensely neglected in previous preclinical and clinical researches. There is an urgent need for novel insights into molecular mechanisms and regimens that uplift the efficacy of immunotherapy since only a minority of cancer patients are responsive to immune checkpoint inhibitors (ICIs). Recent findings on combination therapy of chemotherapy and ICIs have shown promising results. This strategy increases tumor recognition and elimination by the host immune system while reducing immunosuppression by the tumor microenvironment. Currently, several preclinical studies are investigating molecular mechanisms that give rise to the immunomodulation by chemotherapeutic agents and exploit them in combination therapy with ICIs in order to achieve a synergistic clinical activity. In this review, we summarize studies that exhibit the capacity of conventional chemotherapeutics to elicit anti-tumor immune responses, thereby facilitating anti-tumor activities of the ICIs. In conclusion, combining chemotherapeutics with ICIs appears to be a promising approach for improving cancer treatment outcomes.

## Introduction

**Figure 1:**
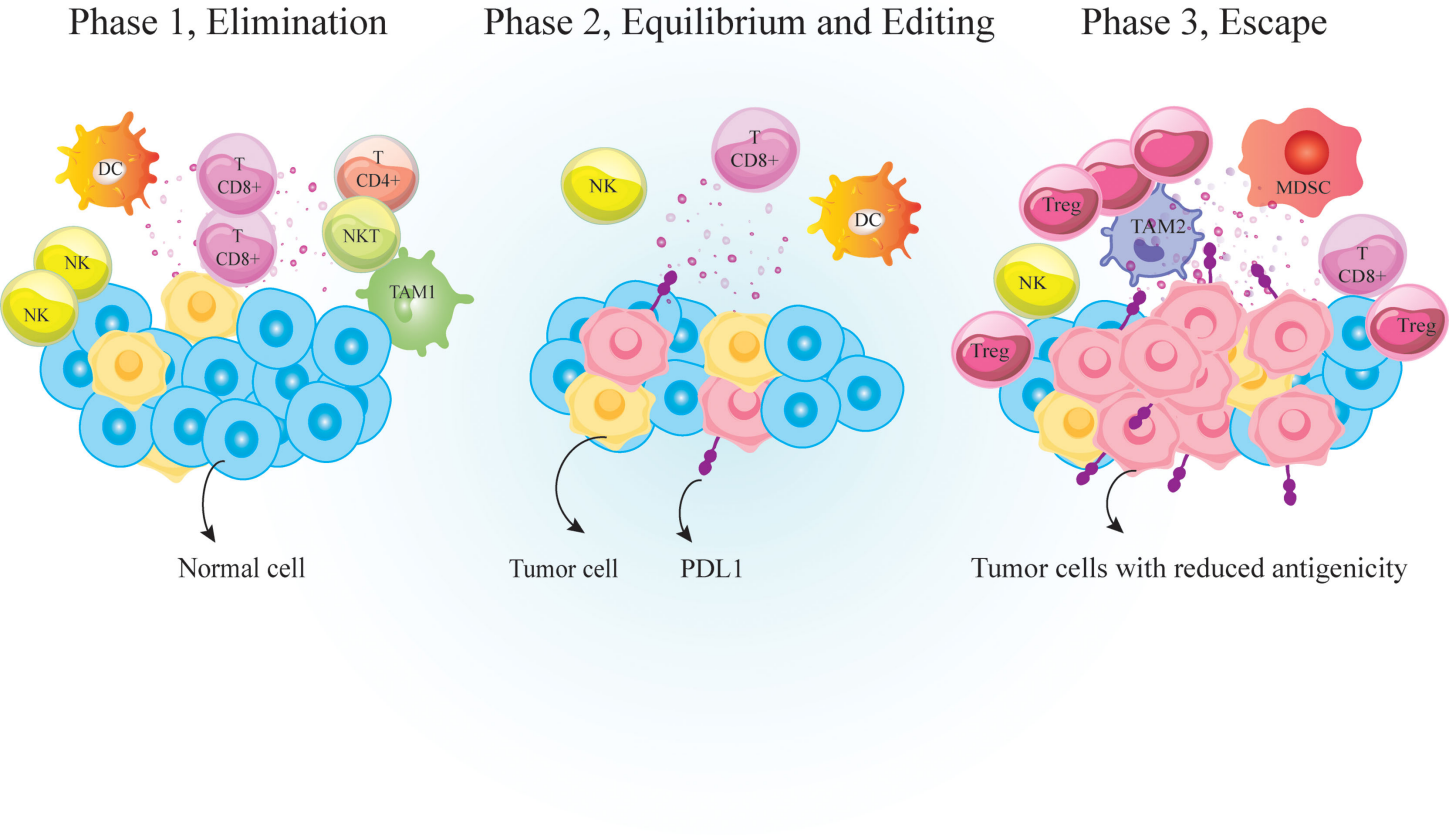
Immunosurveilance and cancer immunoediting. Immunosurveillance is a monitoring process by which cells of the immune system detect and destroy virally infected or malignant cells. It is consisted of three major phases; 1. Elimination phase that eradicate neoplastically transformed cells; 2. Equilibrium phase that occurs upon incomplete eradication of malignant cells so a temporary state of equilibrium develops between the growing tumor and the immune cells; And 3. Escape phase during which variants of tumor cells resist, avoid or suppress the anti-tumor activity of the host immune cells to the point that the immune system is no longer able to restrain tumor growth. PD-1, Programmed cell death 1; TCD4^+^, helper T cell; TCD8^+^, cytotoxic T lymphocyte, DC, dendritic cells; MDSC, myeloid-derived suppressor cell; NK, natural killer; TAM, tumor associated macrophages; Treg, regulatory T-cell.

**Figure 2:**
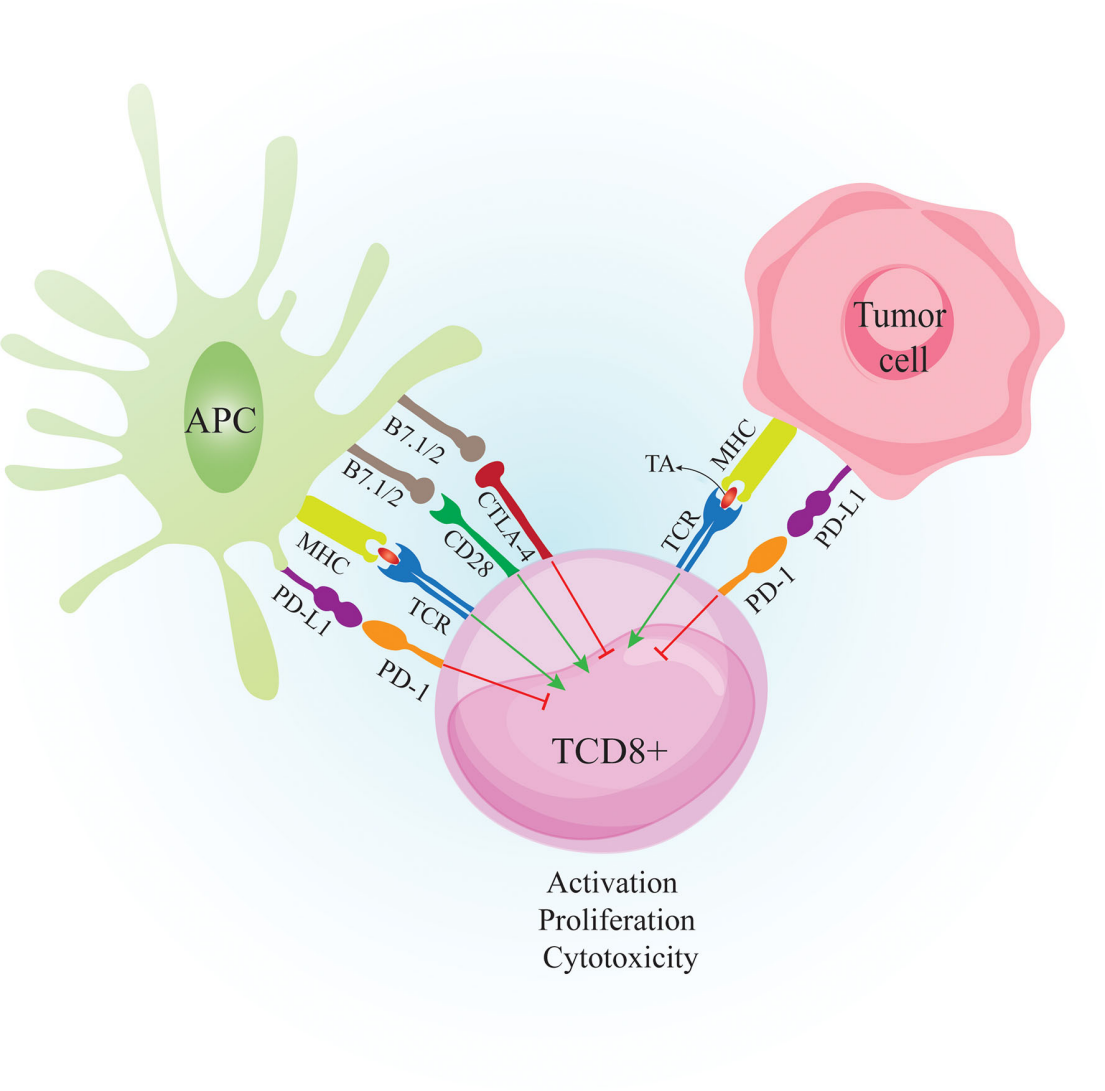
Crosstalk between CTL, APC and Tumor cell. Tumor cells are recognized by the immune system when tumor peptides are presented *via* APC to CD8+ T cells. APC also provide costimulatory molecules such as B7.1/2 to bind CD28 on T cells. The primed CD8+ T cells can recognize tumor antigens and destroy tumor cells by secretion of perforin and granzyme B However, tumor cells express inhibitory molecules such as PD-L1 to bind PD-1 on activated T cells to suppress anti-tumor responses. Tumor cells also induce PD-L1 expression on APCs to further suppress the immune responses. CTLA-4 is expressed on T cells following activation. Binding CTLA-4 on T cells to B7.1/2 on APCs causes inhibition of T cell activity. APC: antigen presenting cell. CTL, cytotoxic T lymphocyte; CD28, cluster of differentiation 2; CTLA-4, cytotoxic T-lymphocyte-associated protein 4; MHC-I, major histocompatibility complex class I; TA, tumor antigen; TCR, T cell receptor; PD-1, Programmed cell death 1; PD-L1, Programmed cell death-ligand 1;.

The link between host immunity and cancer development was unclear until recently. Now it is well documented that cancer development is associated with host immunity. Immunosurveillance is a monitoring process by which immune cells detect and destroy malignant cells (1). Over time, tumor cells evolve to escape immunosurveillance, resulting in tumor establishment (**Figure 1**). Following tumor establishment, cancer cells employ more immunosuppressive mechanisms to escape anti-tumor immune responses (1). Major mechanisms that these cells use for immunosuppression are 1. Binding to effector cells using inhibitory receptors called immune-checkpoints (ICs) such as cytotoxic T-lymphocyte-associated protein 4 (CTLA-4) and programmed cell death protein-1 (PD-1) (**Figure 2**) 2. Secretion of anti-inflammatory cytokines like IL-10 and 3. Suppression of effector cells upon depletion of essential metabolic substrates like tryptophan and arginine (2, 3). So the accumulation of immunosuppressive cells and increased levels of immunosuppressive molecules such as PD-1 ligand (PD-L1) and indoleamine 2,3-dioxygenase 1 (IDO1) are directly associated with poor prognosis and unfavorable disease outcomes in patients with cancer (4–6).

Accordingly, immunotherapeutic approaches such as blocking the ICs and reducing immunosuppressive molecules and cells could enhance the anti-tumor immune responses (7). IC inhibitors (ICIs) such as anti-PD-1 or anti-PDL1 antibodies have shown promising results in the treatment of various cancers like unresectable or metastatic melanoma, renal cancer, metastatic non-small cell lung cancer (NSCLC), and more recently, Hodgkin’s lymphoma and urothelial carcinoma (8–14).

Nevertheless, a significant proportion of patients with breast or prostate cancer show resistance to ICIs, mostly due to the immunosuppressive tumor microenvironment (TME) or the lack of immune checkpoints expression by tumor cells (15–18). Moreover, patients who initially have responded well to ICIs therapy can develop resistance as the disease progresses after a period of time. Therefore, it is essential to prevent resistance phenomena and enhance the anti-tumor activity of monoclonal antibody therapy by using combination therapies. Some studies on combination therapies are ongoing (19–26). Despite the primary belief that conventional chemotherapy is merely immunosuppressive, recent findings unveiled the immunostimulatory properties of chemotherapy. Utilizing chemotherapy leads to the release of antigens through cytotoxic cell death activity, stimulating immune responses and improving the activity of PD-1/PD-L1 blocking agents. In addition, chemotherapy may positively impact the leukocyte composition of infiltrated cells (26–29). Several ongoing clinical trials combine ICIs monoclonal antibodies with various chemotherapies (25–28, 30). This review aimed to discuss the different immunological effects of combining ICIs and chemotherapy.

## ICIs resistance mechanisms and strategies to avoid them

Resistance to immunotherapy drugs can be primary, as seen in non-responders, or acquired, which occurs after some time in patients. Also, resistance can emerge intrinsically or extrinsically. The former happens when tumor cells directly interfere with processes involved in immune recognition, gene expression, and cell signaling. Extrinsic resistance happens externally to tumor cells *via* T-cell activation processes (31).

Several factors in response or resistance to ICIs therapy are related to tumor immunogenicity, TME, antigen presentation, and classic oncologic pathways.

The mechanisms underlying resistance to ICIs are not fully elucidated. However, defects in neoantigen and antigen presentation, mutations in inflammatory signaling pathways, Overexpression of other ICs, and overcoming immunosuppressive mechanisms in the TME can lead to ICI resistance (32).

The defect in antigen presentation is one mechanism of tumor cells to evade immune responses (33). It can stem from DC dysfunction, deficiency in MHC machinery, and decreased T-cell priming. Mutations in B2M (β2-microglobulin gene) lead to MHC-I loss (34). Some acquired ICI-resistant patients showed loss of B2M (35–37). However, not all mutations of B2M cause ICI resistance (32), as it has been reported that some B2M-mutated CRC patients were still ICI sensitive (38).

Co-expression of inhibitory receptors such as Lymphocyte-activation gene (LAG-3), T-cell immunoglobulin and mucin domain-3 (TIM-3), T cell immunoreceptor with Ig and ITIM domains (TIGIT), V-domain Ig suppressor of T cell activation (VISTA) (39), and B-/T-lymphocytes attenuator (BTLA), alteration in the balance of tumor-infiltrating lymphocytes (TILs) in favor of myeloid-derived suppressor cells (MDSCs) and regulatory T cells (Tregs), and increased production of indoleamine 2,3-dioxygenase (IDO) and adenosine are another circumstances that lead to ICI resistance (14, 31).

Neoantigens-specific T cells are principal anti-tumor effector cells that could express ICs (40, 41). Accordingly, loss of neoantigen expression by tumor cells may lead to immune evasion and ICI resistance (42, 43). Neoantigen-specific IC expressing T cell clones might be eliminated by the selective pressure that exists in the TME, resulting in the outgrowth of IC negative clones (44).

Other prominent factors that cause resistance to ICIs are mutations in JAK1/2, IFNGR1/2, and IRF1 (45), aberrant WNT/β-catenin signaling, and loss of tumor suppressor genes (46). Such signalings are required to express ICs on cells (47). Hence, defects in signaling can cause loss of ICs and ICI resistance. However, patients carrying heterozygous mutations still have active signaling pathways and IC expression, making them ICI sensitive (48).

## The rational for ICIs and chemotherapy combination therapy

There are different therapeutic strategies to bypass this resistance to ICIs. Since T lymphocytes are the most effective and crucial components in immune defense against tumor development, any strategy that increases tumor immunogenicity and T cell priming that subsequently activates this type of cell can help defeat tumor resistance. Chemotherapy consists of a large group of molecules that target and destroy proliferating cells. Chemotherapy mainly affects growing cancer cells but may also affect normal proliferating immune cells causing myelosuppression and leukocytopenia. Hence, chemotherapy was long thought to be solely an immunosuppressive treatment modality. However, according to recent studies, there are forms of chemotherapy that demonstrate immune-stimulatory effects (49, 50). Chemotherapy increases tumor response to ICIs therapy by increasing the release of tumor antigens upon cancer cell death, resulting in enhanced T cell priming (29). In addition, chemotherapy contributes to the depletion of the MDSC and Treg population in the tumor site (27). Also, radiation therapy positively affects the ICIs treatment outcome in a similar manner. It increases antigen presentation and promotes an inflamed TME (51). Other strategies available for this purpose that have shown enhanced tumor regression are combining ICI with targeted therapy, cytokine/chemokine inhibitors, and immune-stimulatory agent therapy (31).

Given the complex network of signaling and regulation of immune responses against the tumor, it seems impossible to define a specific immunologic biomarker in order to select patients who would benefit the most from this approach. One of the strategies that help in choosing ICIs therapy alone or in combination with chemotherapy in a cancer patient is related to the immune status of TME and their “hot” or “cold” immunologic contexts. Immunogenic or hot TME comprises infiltrating T cells, inflammatory cytokines, and PD-L1. Contrarily, those lacking these features are called non-immunogenic or cold TMEs (52–55). Patients displaying hot TMEs are excellent candidates for receiving ICI therapy alone, while patients whose tumors are non-immunologic would benefit from the synergistic effect of combination therapies (54). In these patients, chemotherapy boosts immune responses against tumor cells by increasing the immunogenicity of the growing tumor, while IC blockade prolongs this effect by providing a long-lasting immune response resulting in fast tumor regression (56).

## Immunomodulatory effects of chemotherapy

**Figure 3:**
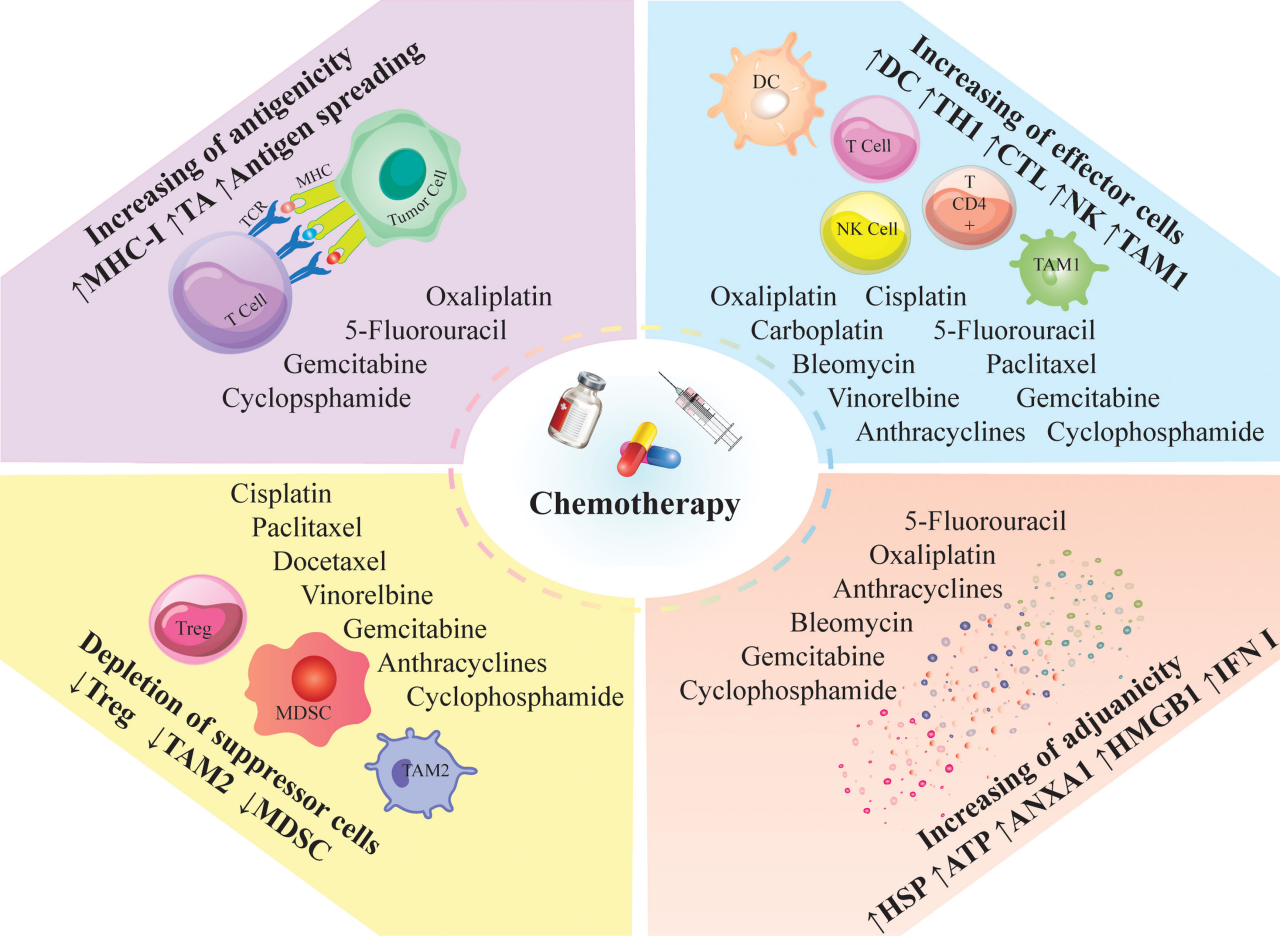
Immunomodulatory effects of chemotherapy; Chemotherapeutic agents can modulate the anti-tumor immune responses; They induce immunologic death and trigger the release of tumor antigens and neoantigens *via* antigen spreading; Besides antigen spreading, they can enhance the release of DAMPs and inflammatory mediators; The induced inflammation can recruit proinflammatory cells into the tumor milieu and decrease the immunosuppressor cells; Such inflammatory conditions; ANXA1, annexin A1; CTL, cytotoxic T lymphocyte; DC, dendritic cells; HMGB1, high mobility group box 1; HSP, heat shock protein; IFN, interferon; MDSC, myeloid derived suppressor cell; MHC-I, major histocompatibility complex class I; NK, natural killer; TAM, tumor associated macrophages; TA, tumor antigen; TCR, T cell receptor; Th, T helper cell; Treg, regulatory T-cell; DAMPs; Damage-associated molecular patterns.

Conventional chemotherapy may exert anti-tumor immune responses by “on-target” effect, which directly increases immunogenicity of targeted cancer cells, or through “off-target” effect on different immune cell populations, leading to alteration of the whole-body physiology favoring anti-cancer immunosurveillance. Common action mechanisms of chemotherapeutic agents are 1. Marked lymphodepletion or so-called ‘reset’ of the immune system (with major adverse effects) and near-to-complete reconstitution of the host’s immunological repertoire (57), 2. Decrease immunosuppressive cells, including M2-like tumor-associated macrophages (TAMs), MDSCs, and Tregs, by providing an inflammatory condition (17, 58, 59), or 3. Activation of effector cells such as cytotoxic T cells (CTLs) (60), DCs (61),, and M1-like TAMS (62) (**Figure 3**).

An efficient anti-tumor immune response relies on the combination of two fundamental factors. First, tumor cells must express antigens for T cell recognition and activation (immunogenicity). Then, cancer cells should recruit adjuvant-like danger signals such as damage-associated molecular patterns (DAMPs) or pathogen-associated molecular patterns (PAMPs) to boost the immunogenicity (adjuvanticity) (63, 64). Conventional chemotherapies improve the immunogenicity and adjuvanticity of cancer cells by inducing cellular death and stress (**Figure 3**) (65).

Immunogenic cell death (ICD), a form of regulated cell death, occurs as a part of the so-called “integrated stress response” and emerges from the activation of unsuccessful cytoprotective pathways such as phosphorylation of eukaryotic translation initiation factor 2 subunit alpha (EIF2S1, also known as eIF2α) and autophagy. Activation of autophagy during ICD leads to the lysosomal secretion of ATP, which promotes purinergic receptors to stimulate the recruitment of DC precursors to the tumor site and accelerates inflammatory reactions after inflammasome activation (66–68). On the other hand, endoplasmic reticulum (ER) stress favors the translocation of ER chaperones such as heat shock protein family A (HSP70) member 1A (HSPA1A, also known as HSP70) and calreticulin to the cell surface. Exposure of these chaperones on the cell surface is a signal for the phagocytic uptake of the tumor cell by immature DCs (69). Accumulation of nuclear or mitochondrial DNA of dying cancer cells in extracellular spaces or the cytosol as a result of responding to chemotherapy elicits immune signaling *via* Toll-like receptor 9 (TLR9), TLR3, and GMP-AMP synthase (CGAS) that eventually results in the production of type I interferons (IFNs) (70, 71). Also, dying tumor cells release a number of nuclear and cytoplasmic proteins like high mobility group box 1 (HMGB1) and annexin A1 (ANXA1). HMGB1 facilitates the interaction of DCs with dead cell debris *via* binding to formyl peptide receptor 1 (FPR1), while ANXA1 promotes DC maturation upon binding to TLR4 (72). These observations represent the importance of adjuvanticity for chemotherapy to exert clinically efficient anti-tumor immunity. Preclinical studies also support this notion by demonstrating the positive prognostic value of the increased DAMP expression and ICD-associated stress responses in the host (73).

However, little is known about the ability of chemotherapy to increase tumor antigenicity. Major histocompatibility complex (MHC) class I upregulation driven by IFN signaling, accumulation of genetic and/or epigenetic defects, and transcriptomic perturbations like modifications in the expression of non-coding RNA are possible mechanisms by which chemotherapy improves antigenicity (74, 75). Supporting this notion, gemcitabine, a chemotherapeutic agent, has been shown to activate stress response pathways that ultimately result in the upregulation of β2 microglobulin (structural component of MHC I) (76).

## Mechanisms of action of chemotherapeutic agents

### Alkylating and platinum based anti-cancer drugs

Alkylating agents can stop protein synthesis by inhibiting the transcription of DNA into RNA. Anti-tumor alkylating agents covalently bind to the double strand of DNA and form platinum–DNA crosslinks. These inter- and intra-strand crosslinks disrupt DNA replication and transcription and ultimately induce apoptosis in cancer cells. In addition, platinum agents can exert their anti-tumor efficacy by modulating the host’s immune system. Cisplatin, oxaliplatin, and carboplatin are renowned platinum derivatives. Moreover, cisplatin is the most studied derivative regarding immunomodulatory effects (77–79). Oxaliplatin and cisplatin increase type I IFNs and IFN-γ signaling, leading to the upregulation of co-inhibitory ligands such as PD-L1 (80). Similarly, it has been observed that chemotherapy consisting of platinum-containing agents and 5-fluorouracil (5-FU) induces PD-L1 expression as a consequence of enhanced CD8+ CTL infiltration (81). Also, the same effect has been reported in patients with esophageal squamous cell carcinomas who received cisplatin plus 5-FU (82).

Platinum agents decrease STAT6 signaling through dephosphorylation of STAT6. Loss of STAT6 phosphorylation leads to the downregulation of PD-L2 on tumor cells and DCs and, in turn, increases tumor recognition by T lymphocytes (83, 84). Also, platinum agents can stimulate CTL-mediated attack (83). A combination of cisplatin and vinorelbine in patients with NSCLC made tumor cells more responsive to MHC-guided perforin and granzyme-B mediated CTL attacks, mainly associated with the MHC I upregulation (85). Besides this mechanism of action, it was reported that combining cisplatin with vinorelbine may increase the ratio of effector CD4^+^ to Tregs (86). However, cisplatin is unable to stimulate calreticulin release from ER, thereby not able to completely trigger ICD (87). In the same context, carboplatin is inefficient in inducing ICD, resulting in the partial release of HMGB1 and calreticulin. Conversely, platinum compounds play an important role in recruiting and activating DCs to tumor sites by inducing the release of ATP from dying cells (83). However, further investigations are required to elucidate the exact effect of various chemotherapeutic compounds on the immune response.

Cyclophosphamide was the first chemotherapeutic agent reported to have immunomodulatory effects, if used at a certain dosage, by selective depletion of the Treg population. This observation initiated the investigation of the probable immunomodulatory effects of other agents (88). Furthermore, cyclophosphamide is able to trigger DCs homeostasis. Supporting this notion, mouse models treated with intraperitoneal cyclophosphamide showed tumor cell death with tumor infiltration and engulfment of apoptotic tumor cells by DCs, and ultimately cross-priming of CD8+ T cells by DCs (89). Other *in vivo* experiments have demonstrated that a single dose of cyclophosphamide converts the immune profile from TH2 to TH1 cytokines, increasing interleukin (IL)-2 and IFN-γ production and decreasing IL-10 and TGF-β production (90–92). Mouse Tregs appear to be less sensitive to cytotoxic effects of cyclophosphamide compared with human Tregs. Nevertheless, systemic cyclophosphamide mediates IL-12 and interferon regulatory factor 1 (IRF1)-dependent response resulting in increased TH1 polarization and Treg depletion (93). It is unclear whether a similar pathway is operational in humans. Another mechanism of anti-tumor efficacy of cyclophosphamide is inducing ICD (94).

The combination of cyclophosphamide and oxaliplatin in NSCLC patients significantly increased nuclear HMGB1 staining in tumor nodules; Also, the oxaliplatin cyclophosphamide combination was able to control tumor growth (95). It is important to mention that the dosage of cyclophosphamide used in combination therapies is crucial: high dosages may induce myelosuppression, while metronomic low dosages improve the immune system (96, 97). Low-dose cyclophosphamide has been observed to deplete both circulating and tumor-infiltrating Treg cells through ICD-independent mechanisms (98, 99). Of note, cyclophosphamide depletes both Treg cells and TH1 cells at first; however, TH1 cells are able to recover faster than Tregs after discontinuation of the treatment (100).

### Topoisomerase inhibitors

Topoisomerase I and II are normal enzymes of mammalian cells that cut and repair DNA strands in DNA replication and cell division processes. Their activity significantly increases in rapidly dividing cancer cells. Topoisomerases represent an appropriate non-selective anti-cancer drug. Camptothecin is an active topoisomerase poison, and its derivatives, irinotecan and topotecan, serve as topoisomerase I inhibitors (101). According to preclinical findings, camptothecin derivatives enhance tumor recognition by T cells. Also, topoisomerase I inhibitors increase the expression of TP53INP1 and Melan-A/MART-1. Upon overexpression of these antigens, tumor recognition by T lymphocytes is increased, and consequently, T cell-mediated killing of cancer cells is improved (102, 103). Another study demonstrated upregulation of HMGB1, HSP70, and other DAMPs after treatment with irinotecan (104). MHC I and Fas expression are upregulated in tumor cells affected by topotecan treatment, making them more sensitive to killing by effector T cells (105, 106).

Anthracyclines are inhibitors of topoisomerase II and have shown to be effective in the selective depletion of immunosuppressive cells. Doxorubicin, epirubicin, and idarubicin are derivates of anthracyclines with immunosuppressive abilities (82). Clinical results in patients with breast cancer demonstrated that intraperitoneal administration of 5mg/kg doxorubicin leads to *in vivo* reduction of MDSCs and subsequent increase of CD4+ and CD8+ T cells, as well as other effector elements including IFN-γ, granzyme B and perforin (107). Besides their immunosuppressive capabilities, anthracycline has the capacity to elicit ICD by increasing the expression of DMAPs, including HMGB1, HSP70, and calreticulin (72, 108). However, inducing ICD requires higher doses than cytotoxic dose (109).

Other immunomodulatory effects of anthracyclines have been studied, and it appears that they can induce immune responses in a similar manner that viral pathogens initiate immune responses (71). Doxorubicin acts by damaging DNA and cell membrane by generating free oxygen radicals. It demonstrated limited clinical efficacy in NSCLC treatment mainly due to the rapid upregulation of NF-KB signaling in response to therapy and acquiring resistance in the host immune system (110). Doxorubicin can also exert anti-tumor activity by upregulating STAT1 signaling (78, 87). Epirubicin disrupts Treg-mediated suppression of CD8+ T cells by blocking the interaction between NF-KB subunit p65 and Foxp3 *in vitro* (111). A few studies have reported potential negative outcomes of anthracyclines. Daunorubicin has been observed to induce cell death in both resting and activated peripheral blood cells, which is considered a negative factor for ICI combination (112, 113).

### Antimitotic agents

Anti-microtubule agents exert anti-neoplastic effects by disrupting microtubules. The most widely used anti-microtubule agents are Taxanes: Docetaxel and paclitaxel (114). Taxanes are generally known for depleting neutrophils and lymphocytes, especially neutrophils (115). Increased neutrophil to lymphocyte ratio favors ICIs treatment (116). CD3^+^, CD4^+^, CD8^+^, CD56^+^, and CD45RO+ cells are lymphocytes affected by taxane administration (117), some of which are related to ICI responses (118).

Taxanes improve the upregulation of proinflammatory cytokines such as IL-2, IL-6, IFN-γ, and GM-CSF after six cycles of standard treatment (119). The effect of taxane agents on the cytotoxicity of T cells is controversial. While some studies report impaired T cell-mediated cytotoxicity upon treatment with paclitaxel (120), others have shown increased NK and lymphokine-activated killer cell activity (119, 121). Moreover, both agents have immunomodulatory effects on specific immune cell subsets (79). At certain dosages, paclitaxel and docetaxel induce DC maturation by increasing the expression of MHC II (122). Also, it reduces the Treg population through upregulation of Fas receptors and increasing apoptosis while improving CD4^+^ and CD8^+^ T cells (123). Similarly, docetaxel exerts its anti-tumor activity by selective depletion of Tregs and reduction of MDSCs at the tumor site *via* activation of STAT3 signaling (56, 124).

Administration of a chemotherapeutic cocktail containing doxorubicin and cyclophosphamide contributes to the repolarization of the TME compartment from an M2-like phenotype to an M1-like phenotype (125). A similar finding was observed upon administrating paclitaxel which is reported to act as a TLR4 agonist (62). According to preclinical data, paclitaxel supports the upregulation of co-inhibitory ligands like PD-L1 by tumor-infiltrating myeloid cells or malignant cells as a consequence of type I IFN or IFN-γ signaling (126, 127). Therefore, upregulation of PD-L1 upon chemotherapy is used as a biomarker to classify and select patients who would benefit from anti-PD-1 or anti-PD-L1 antibodies alone or in combination with chemotherapy (128).

One of the concerns regarding the administration of chemotherapeutic agents is adverse effects like neuropathic pain and neuroinflammation. According to recent preclinical findings, gut microbiota can modulate neuroinflammation induced by taxanes (129, 130). Thereby, modulating gut microbiota by using prebiotics or probiotics may enhance the efficacy of chemotherapy and help manage the toxicities of these agents (131).

### Antimetabolites

Antimetabolites are chemotherapeutic agents with ‘cytotoxic’ effects on cells by mimicking the molecules, such as genetic materials and enzymes that tumor cells need for growth. Thereby, tumor cells uptake and use these antimetabolites instead of normal cell materials (132).

Gemcitabine is an antimetabolite and pyrimidine analog that disrupts RNA and DNA synthesis (133). It modulates anti-cancer responses by selectively suppressing MDSCs (134) while increasing the expression of tumor antigens and making tumor cells more recognizable to the immune system. In a trial on patients with pancreatic cancer, the standard dosage of gemcitabine resulted in Treg depletion that lasted for two weeks (135). 5FU is extensively used as an anti-cancer drug. Since 1957, it has been important in treating cancers like colon cancer and breast cancer (128, 129). 5-FU has a structure similar to the pyrimidine molecules of DNA and RNA and is a uracil analog. It interferes with nucleoside metabolism and can be incorporated into RNA and DNA, leading to cytotoxicity and cell death (130). A standard dosage of 5FU exerts stimulatory effects by supporting antigen uptake of DCs. In an *in vitro* study, a gastric cancer cell line pre-treated with 5FU showed higher IL-12 production than the control. This subsequently increased the cytotoxicity of T cells generated by DCs compared to the control (136). Both gemcitabine and 5FU activate the NLRP3 inflammasome in MDSCs, leading to IL-1β secretion and ultimately immunosuppression by TH17 (137). In CT26 tumor-bearing mice, 5FU eliminated MDSCs without significantly affecting T, B, or NK cells (138).

Methotrexate is a folate derivative that can inhibit a number of enzymes involved in nucleotide synthesis culminating in the suppression of inflammation and prevention of cell division. High-dose methotrexate suppresses bone marrow, while low-dose methotrexate has been reported with immune-stimulating properties (139). According to an *in vitro* study, low-dose methotrexate supported DC maturation by upregulating CD40, CD80, and CD83. Consequently, DCs stimulated T cell proliferation and ultimately exerted a proper anti-tumor immune response (140).

Pemetrexed is another antimetabolite that inhibits enzymes involved in the folate pathway, including thymidylate synthase, dihydrofolate reductase, and glycinamide ribonucleotide formyltransferase (141–143). Despite the fact that the folate pathway is critical for T cell activation, several studies have reported improved T cell infiltration and antigen presentation in tumor sites upon pemetrexed administration (144). In addition, pemetrexed selectively activates CD45RO^+^ memory T cells and IFN-γ-producing NK cells (84).

## Clinical trials of ICIs and chemotherapy combination therapy

Immunotherapy, particularly ICIs that target PD-1, PD-L1, and CTLA-4, is being widely used and becoming the predominant treatment modality in patients resistant to conventional therapies. ICIs generate moderate-to-severe side effects that require immunosuppressive drugs and active clinical management in some patients (145). Therefore, extensive research has been done in order to develop a proper combination therapy of chemotherapy and ICIs therapy to achieve early (with chemotherapy) and long-lasting (with ICIs) disease control while yielding superior overall survival and minimum risk of adverse effects.

Noteworthy, besides the augmenting effects of chemotherapy on ICI responses, ICIs can also enhance the clinical efficacy of chemotherapy in a reciprocal way (146). Chemotherapy increases the infiltration of immune cells in the tumor. However, a significant proportion of the infiltrated immune cells express ICs over time, limiting their functions (7, 63). Hence, adding ICIs to chemotherapy could augment the responses to chemotherapy (146).

**Table 1** demonstrates trials using ICI-chemo combination therapy. In a phase II clinical trial in NSCLC patients, KEYNOTE-021, the efficacy of combining anti-PD-1 (pembrolizumab) with chemotherapy was investigated. It was observed that the combination of anti-PD-1 with carboplatin and pemetrexed culminated in a higher response rate than chemotherapy alone (180). However, adding immunomodulation to chemotherapy did not lead to enhanced toxicity (180, 181). An updated analysis confirmed improved response rate and progression-free survival of this combination therapy which led to its accelerated FDA approval for treatment of metastatic NSCLS patients. Interestingly, the enhanced response rate of 80% was associated with higher tumor PD-L1 expression (≥50%).

**Table 1:** Different clinical trials using combination therapy.

Tumor Type	Chemotherapy	Immunotherapy	Findings	Reference
All solid tumor types	Docetaxel Nab-paclitaxel Gemcitabine Vinorelbine Irinotecan liposomal oxorubicin	Pembrolizumab	Full dosages of chemotherapy were used. Phase 2 dose was found to be as maximum tolerated dose. Partial responses occurred in arm 3 – 6.	(147)
Breast cancer (Triple negative)	Gemcitabine Carboplatin	Pembrolizumab	In two out of three patients effective immune stimulation observed	(148)
Breast cancer (Triple negative)	Eribulin	Pembrolizumab	Median PFS 4.2 mo OS 17.7 mo	(149)
Breast cancer (Triple negative)	Capecitabine or Paclitaxel	Pembrolizumab	Three out of nine patients showed a partial response although two patients had metastatic disease.	(150)
Breast cancer (Triple negative)	Doxorubicin Paclitaxel Cyclophosphamide Carboplatin	Pembrolizumab	In both regimens promising anti-tumor activity observed. Addition of carboplatin resulted in more grade 3 or 4 toxicities, mainly neutropeni.	(151)
Breast cancer (Triple negative)	Carboplatin	Nivolumab	NR	(152)
Breast cancer (Triple negative)	Eribulin	Durvalumab	NR	(153)
Breast cancer (Triple negative)	Nab-paclitaxel Cyclophosphamide Epirubicin	Durvalumab	Combination therapy resulted in a high CR rate and induction therapy with durvalumab seemed useful.	(154)
Breast cancer (Triple negative)	Nab-paclitaxel	Atezolizumab	Median PFS 5.5 mo OS 14.7 mo	(155)
Breast cancer (HER2 negative)	Paclitaxel	Nivolumab	NR	(156)
Colon cancer	5-Fluorouracil Oxaliplatin	Durvalumab, Tremelimumab	Phase 1b: Safety Phase 2: Primary: PFS Secondary: OS	NCT03202758 (phase 1b/2)
Gastric cancer	Paclitaxel	Nivolumab	NR	(157)
Gastric cancer	Cisplatin 5-Fluorouracil	Pembrolizumab	Effective immune stimulation observed in Full dose administration irrespective of PD-L1 expression	(158)
Head and neck cancer	Cisplatin	Avelumab	NR	(159)
Mesothelioma	Cisplatin Pemetrexed	Nivolumab	NR	(160)
Melanoma	Dacarbazine	Ipilimumab	ORR was 14.3% vs 5.4% for the combination therapy. OS was 20.9 and 16.4 respectively	(161)
Melanoma	Dacarbazine	Ipilimumab	Combination therapy resulted into a higher OS (11.2 movs. 9.1 mo).	(162)
Melanoma	Carboplatin Paclitaxel Temozolomide Nab-paclitaxel	ICIs	Patients who received chemoimmunotherapy had a median OS of 5 years (95% CI: 2-NR) versus 1.8 years (95% CI: 0.9-2; p = 0.002) for those who received either ICIs or chemotherapy alone, with ORR of 61% versus 17% (p = 0.001), respectively	(163, 164)
NSCLC	Carboplatin Nab-paclitaxel	Pembrolizumab	Approved for first line treatment of metastatic squamous NSCLC. Improved OS (15.9 mo vs 11.3 mo), response rates, and duration of response (PFS if 6.4 mo vs 4.8 mo) in the group with chemoimmunotherapy compared to chemotherapy alone.	(165)
NSCLC	Pemetrexed Cisplatin Carboplatin	Pembrolizumab	Pembrolizumab combination group: OS (12 mo): 69.2% PFS: 8.8 m Placebo combination group: OS (12 mo): 49.4% PFS: 4.9 mo	(166)
NSCLC	Pemetrexed Carboplatin	Pembrolizumab	Pembrolizumab + Pemetrexed+ Carboplatin: ORR: 56.7% PFS: 24.0 mo Pemetrexed/Carboplatin: ORR: 30.2% PFS: 9.3 mo Approved for first line treatment of metastatic non-squamous NSCLC.	(167, 168)
NSCLC	Cisplatin Gemcitabine Pemetrexed or cisplatin	Nivolumab	NR	(169)
NSCLC	1: Gemcitabine + cisplatin 2: Pemetrexed + cisplatin 3: Paclitaxel + carboplatin	Nivolumab	Group 1 PFS: 5.7 mo OS: 11.6 mo Group 2 PFS: 6.8 mo OS: 19.2 mo Group 3 (With 10 mg/kg nivolumab) PFS: 4.8 mo OS: 14.9 mo Group 3 (With 5 mg/kg nivolumab) PFS: 7.1 mo Most promising results observed in group 3	(170)
NSCLC	Gemcitabine Cisplatin Paclitaxel Carboplatin Pemetrexed Docetaxel	Nivolumab	NE	(171)
NSCLC	Paclitaxel Carboplatin	Ipilimumab	Ipilimumab + Carboplatin + Paclitaxel: OS: 13.5 mo PFS: 5.6 mo Placebo + Carboplatin + Paclitaxel: OS: 12.4 mo PFS: 5.6 mo	(172)
NSCLC	Paclitaxel Carboplatin	Ipilimumab	Only phased regimen leads to improved PFS compared to control	(173)
NSCLC	Carboplatin Bevacizumab Paclitaxel	Atezolizumab	Approved for first line treatment of metastatic non-squamous NSCLC with atezolizumab + bevacizumab + chemotherapy	(174)
NSCLC	Carboplatin Etoposide	Atezolizumab	Atezolizumab group: OS: 12.3 mo PFS: 5.2 mo Placebo group: OS: 10.3 mo PFS: 4.3 mo	(175)
NSCLC	Carboplatin Paclitaxel Pemetrexed Nab-paclitaxel	Atezolizumab	Atezolizumab+ Carboplatin + Paclitaxel ORR: 36% PFS: 7.1 months OS: 12.9 months Atezolizumab + Carboplatin/Pemetrexed ORR: 68% PFS: 8.4 mo OS: 18.9 mo Atezolizumab+ Carboplatin+ Nab-paclitaxel: ORR: 46% PFS: 5.7 mo OS: 17.0 mo	(176)
STS	Trabectidin	Nivolumab	Synergistic and safe effect in paired administration of trabectedin and nivolumab	(177)
Urothelial cell carcinoma	Gemcitabine Cisplatin	Ipilimumab	No changes was observed in composition and frequency of peripheral immune cells upon gemcitabine administration. Expansion of CD4+ cells occurred after combination therapy.	(178)
Urothelial cell cancer	Docetaxel or gemcitabine	Pembrolizumab	Arm A ORR: 50%, DCR: 67%, PFS: 5.7 mo Arm B: ORR: 33%, DCR: 50%, PFS: 3.7 mo	(179)

CR, complete response; DCR, disease control rate; ICIs, immune checkpoint inhibitors; MTD, maximum tolerated dose; NSCLC, non small cell lung cancer; ORR, objective response rate; OS, overall survival; PFS, progression free survival; STS, soft tissue sarcoma; mo, months; NR, not reached; NE, not evaluable;

Subsequently, the KEYNOTE-189 phase III trial evaluated the efficacy of adding anti-PD-L1 therapy to chemotherapy in non-squamous NSCLC (182). Results indicated improved overall survival and progression-free survival compared to chemotherapy alone, independently of the tumor PD-L1 status. This culminated in full FDA approval for the combination approach of pembrolizumab and chemotherapy in treating NSCLC patients. Nevertheless, these suggestions are subject to change upon approval of nivolumab and ipilimumab for patients with PD-L1- positive tumors (PD-L1 ≥1%) and in combination with chemotherapy regardless of PD-L1 expression (183, 184). A phase III trial (KEYNOTE-407) is ongoing to evaluate whether this combination can also benefit patients with squamous NSCLC. Overall, adding ICI to conventional chemotherapy has modified the standards for treating metastatic lung cancer patients (182).

Despite extensive research on combining ICIs with chemotherapy, only a few trials attempted to systematically determine the most effective chemo-ICIs immunotherapy. The TONIC trial is the only trial to assess the possibility of sensitizing patients with metastatic breast cancer after administrating a PD-1 inhibitor and a chemotherapy agent. Five therapeutic options were tested: no treatment, radiotherapy, cisplatin, cyclophosphamide, or doxorubicin. Doxorubicin appeared to have the strongest sensitizing effect (185). It should be noted that this was a small-scale trial aiming to compare the efficacy of a few induction regimens, including systemic chemotherapy or local radiotherapy treatment in metastatic cancer (186). Thus, the results of this particular should not be generalized to other malignancies and interpreted as definite evidence regarding the superiority of doxorubicin over other induction therapies. Other studies have also used PD-1 inhibitors in combination with chemotherapy. Camrelizumab is a high-affinity, fully humanized, anti-PD-1 IgG4 type monoclonal antibody that blocks the binding of PD-1 to its ligands (187).

Fang et al. have published initial data for a nonrandomized single-arm phase I trial investigating camrelizumab as a first-line treatment for patients with metastatic nasopharyngeal carcinoma. The study investigated gemcitabine and cisplatin combined with camrelizumab (followed by maintenance camrelizumab). More of interest, the combination of camrelizumab with gemcitabine and cisplatin had good clinical efficacy with 20 (ORR 91%) out of 22 patients achieving an overall response after a median follow-up of 10.2 months (188).

In phase III clinical trial (PACIFIC trial) in patients with unresectable NSCLC, anti-PD-L1 antibody durvalumab was administered with chemotherapy plus radiotherapy. Improved treatment outcomes were observed with remarkable improvement in overall survival compared to placebo (median time to death or development of distant metastases 28.3 versus 16.2 months) (183). The same results were achieved in a trial in cohorts of patients with metastatic NSCLC who received anti-PD-L1-antibody atezolizumab combined with paclitaxel, bevacizumab, and carboplatin (184). In addition, both durvalumab and atezolizumab were found to be efficient in monotherapies in patients with metastatic NSCLC (189, 190). This supports the notion that the positive therapeutic performance of these combination therapies may be simply related to the effect of anti-PD-1 monoclonal antibodies.

A negative point of current clinical trials that use combination therapy is that the majority of them administer chemotherapy and ICIs concomitantly at full doses. A study evaluated three different regimens using the combination of ipilimumab and gemcitabine in non-immunogenic mouse models (191). Gemcitabine was used 15 days prior to anti-CTLA-4, concurrently, and three days after anti-CTLA-4. The results showed synergistic effects in the concomitant regimen and removing the first dosage of gemcitabine significantly reduced anti-tumor effects. Similar results were found in another *in vivo* study that combined cyclophosphamide and anti-CTLA-4 (192). Administration of cyclophosphamide one day prior to anti-CTLA-4 improved immunological anti-tumor responses. However, when the orders were reversed, the anti-tumor effects of anti-CTLA-4 were decreased, and CD8^+^ T cells underwent massive apoptosis. These findings prove that chemotherapy accentuates the anti-tumor effects of ICI therapy. Nevertheless, only a few studies have addressed the optimal dosage or sequence of administration of chemotherapeutic agents. Preclinical data shows that these parameters may affect treatment outcomes.

It has been reported that the induction phase of chemotherapy may optimize TME for the following ICIs therapy. A study in metastatic triple-negative breast cancer patients evaluated induction therapy with different types of chemotherapy. In the induction phase, low dosages of cisplatin, doxorubicin, and cyclophosphamide were administered for two weeks. In a cohort receiving this regimen, the response rate appeared to be higher than nivolumab alone (193). By far, favorable response rates have been observed upon induction of doxorubicin and cisplatin. Biomarker analysis also supported the notion that treatment with these two agents culminates in the upregulation of immunological pathways related to anti-PD-1, which ultimately facilitates the nivolumab anti-tumor effect. In addition, inducing these two compounds increased TILs in TME (194).

Another phase II clinical trial investigating the performance of ipilimumab with paclitaxel and carboplatin was conducted in NSCLC patients. Three different regimens were evaluated: Administration of chemotherapy prior to ipilimumab, concomitant regimen, and the control group receiving placebo and chemotherapy. Similar to previous trials, results indicated the importance of the chemotherapy induction phase (173). Thus far, most of these trials provided inconclusive preclinical evidence regarding the optimal dosage and sequel of administration. Therefore, a large multi-center trial is needed to determine the optimal combinations of ICIs and immunogenic chemotherapy for cancer treatment.

## Dose and time optimization for ICIs and chemotherapy combination therapy

It should be noted that chemotherapy might impose immunosuppressive effects based on dose. High doses of cytotoxic chemotherapy have myelosuppression effects, leading to immunosuppression and ICI resistance (146). Moreover, high-dose chemotherapy causes off-target effects and toxicity. However, it has been implied that the doses for induction of immunologic cell death are generally higher than the cytotoxic doses of chemotherapy (109). So, optimum doses of chemotherapy are required to enhance the ICIs response. Interestingly, a study showed that daily prescription of 100 mg oral cyclophosphamide decreased Treg proportion without significant effects on other immune cells. However, doubling this dose depleted all lymphocytes (97). This study concluded that the metronomic doses of cyclophosphamide could decrease Tregs and spare effector T and NK cells (97).

Besides the treatment dose, treatment time is also critical in achieving the best outcome. It has been reported that a single standard dose of gemtacibine increased the infiltration of CD8+ T cells into the tumor and upregulated the PD-L1 expression in pancreatic and ovarian tumors (127, 135). However, this effect is observed within the first week and not in the second week after treatment (127). So, administration of ICIs within the first week of treatment with gemtacibine might result in favorable responses. Accordingly, similar studies on mice showed that ipilimumab had a synergistic effect with gemtacibine or cyclophosphamide only when administered concomitantly (191, 192). Interestingly, anti-tumor immune responses are yielded when chemotherapy is administered one day before the ICI. However, reversing the order caused significant apoptosis in CD8+ T cells and reduced the ICIs effects (192). These findings indicate the necessitate to optimize the dose and timing of treatment.

Patient-derived organoid (PDO) platforms are promising models to optimize the dose and time of treatment ex vivo (195). Advanced PDOs, such as air-liquid interface (ALI) models that contain both tumor and immune cells in a 3D interaction resembling tumor milieu, can be used in further investigations to determine the optimum dose and time and also the ideal combination of chemo-immunotherapy (196, 197).

## Combination of chemotherapy with other immunotherapies

According to the systemic and local changes that chemotherapy makes, it can synergize the effects of many immunotherapies. Adoptive cell therapy is an immunotherapeutic approach that uses the patient’s or another donor’s immune cells to fight cancer (18). Tumor-infiltrating lymphocytes (TILs) are immune cells infiltrating into the TME (17). The frequency of TILs in the TME is a prognostic factor in many tumors (198). Isolation, expansion, activation, and re-administration of TILs to the patients is an old way of immunotherapy in immunogenic tumors (17). However, in non-immunogenic or cold tumors with a low frequency of TILs, TIL therapy might not significantly improve the survival (17). In this condition, chemotherapy might heat up the TME, leading to a higher infiltration rate of TILs (199). So, it can potentiate TIL therapy in cold tumors (199). More recently, CAR-T cell therapy has been introduced and used as a promising anti-tumor modality (200). CAR-T cells are genetically modified T cells that harbor the chimeric antigen receptors comprising the extracellular domain of B cell receptor (BCR) and signaling domains of T cell receptors (TCRs) and co-stimulatory receptors (200). They can be activated independent of MHC and produce many cytokines or release cytotoxic molecules (200). Chemotherapy can enhance the susceptibility of tumor cells to cytotoxic mediators of T cells such as granzyme B (201). In the mouse model of breast cancer, combining doxorubicin and T cell therapy had synergistic effects beyond the effects of each treatment alone (202). Further clinical trials in the era of combining chemotherapy and adoptive cell therapy are warranted.

Chemotherapy is also beneficial for improving the effects of cancer vaccines, especially in cold tumors (203–205). In a mouse model of melanoma (B16), it has been shown that the addition of ICI (anti-PD-1) to the cancer peptide vaccine did not further inhibit the tumor growth and was not able to improve the survival (203). However, combining metronomic chemotherapy with the vaccine resulted in tumor growth inhibition in half of the mice (203). Notably, adding ICI to this combination delayed tumor growth in all mice and inhibited tumor growth in two-thirds of mice (203). This synergistic effect of chemotherapy with cancer vaccines might be through the decrease of Tregs and inhibition of tumor angiogenesis (204). These findings suggest that multi-aspect combination therapy should be employed in advanced and resistant tumors.

Oncolytic viruses (OVs) are wild-type or engineered viruses with anti-tumor capabilities that is able to impose direct cytotoxic or heat up the TME *via* upregulation of the inflammatory responses (206). Similar to chemotherapy, OVs can induce ICD, releasing neoantigens and inflammatory mediators that potentiate the immunotherapies (207). However, chemotherapy and OVs can be combined to maximize the inflammatory and cytotoxic responses against tumor cells (207). There are many clinical trials evaluating the safety and efficacy of chemo-OV combination therapy that are comprehensively reviewed elsewhere (207).

Chemotherapy can be combined with many other immunotherapies and targeted therapies, such as monoclonal antibodies, bispecific antibodies, nanobodies, DC vaccines, NK/CAR-NK cells, etc., which their safety and efficacy of these combinations are under investigation.

## Perspectives and conclusions

According to several clinical trials, chemotherapeutic agents can exert immunostimulatory effects by activating effector cells and/or inhibiting immunosuppressive cells or elevating immunogenicity and enhancing T-cell infiltration. However, more research is required to achieve the best and most efficacious combination therapy. In order to do so, it is suggested that future researches focus on the following suggestions: 1. Preclinical studies that evaluate drug efficacy should be compatible as much as possible with the clinical situation. Thereby, results obtained from animal models can also be utilized for human malignancies. 2. Until now, most studies assessed the immunomodulatory effects of chemotherapy in peripheral blood and not TME. Further studies investigating the effect of chemotherapy on TME are required. 3. In addition to chemotherapy, ICD can also be induced as a result of pathological conditions. Therefore, it is crucial to differentiate between chemotherapy-induced ICD and the type of ICD caused by normal physiological processes and other pathological conditions. 4. Identifying tumor-associated markers specific to each tumor and each cancer patient may help design better combination therapies in the future. 5. Furthermore, understanding the toxicity patterns of these treatment regimens in preclinical studies would provide us with more knowledge of how to prevent and manage them in clinical studies. In conclusion, various clinical and preclinical findings indicate that combination therapy can result in a more efficient, long-lasting anti-tumor immune response.

## Author contributions

WL, conception, design, and inviting co-authors to participate. LZ, CZ, SZ, and XC, writing original manuscript draft. WL, JL, and FX, review and editing of manuscript critically for important intellectual content and provided comments and feedback for the scientific contents of the manuscript. All authors contributed to the article and approved the submitted version.

## Funding

This work was supported by the Project of Shaoxing Medical Key Discipline Construction Plan (2019SZD06 to LZ) and the Project of Health and Family Planning Commission of Zhejiang Province (2021KY1139, and 2018KY831 to LZ), Shaoxing Medical and Health Science and Technology Plan Project (2020A13026 to LZ).

## Conflict of interest

The authors declare that the research was conducted in the absence of any commercial or financial relationships that could be construed as a potential conflict of interest.

## Publisher’s note

All claims expressed in this article are solely those of the authors and do not necessarily represent those of their affiliated organizations, or those of the publisher, the editors and the reviewers. Any product that may be evaluated in this article, or claim that may be made by its manufacturer, is not guaranteed or endorsed by the publisher.
